# Giant Cell Tumour Around Knee Managed by Curettage and Zoledronic Acid with Structural Support by Fibula Cortical Struts

**DOI:** 10.5704/MOJ.2011.008

**Published:** 2020-11

**Authors:** V Singaravadivelu, V Kavinkumar

**Affiliations:** Institute of Orthopaedics and Traumatology, Rajiv Gandhi Government General Hospital, Chennai, India

**Keywords:** GCT, zoledronic acid, fibula strut, recurrence

## Abstract

**Introduction:**

Giant cell tumour (GCT) of the bone is a benign tumour with a high tendency to recur after surgery. This study aimed to analyse prospectively the rate of local recurrence following management of giant cell tumours by curettage, using intravenous zoledronic acid as an adjuvant, and fibular struts to support the empty cavity after curettage.

**Materials and Methods:**

This study was carried out in ten cases of biopsy-proven GCTs: five males and five females, in the age group between 18 and 39 years. All patients were given three doses of zoledronic acid, one pre-operative and two post-operative. Extended curettage was done three weeks after the pre-operative dose of zoledronate. The cavity was left empty in all the cases. Fibular struts were used to support the cavity from collapse. Patients were followed-up for post-operative local recurrence. The functional status of the patients was assessed during each visit using the Musculoskeletal Tumour Society (MSTS) score.

**Results:**

There were no recurrences at a follow-up of two years. All patients had a stable knee and were able to bear weight fully. The average knee flexion was 75º. The average MSTS score of the study was 92%.

**Conclusion:**

Extended curettage using hydrogen peroxide, systemic zoledronic acid adjuvant and leaving the cavity empty without using cancellous bone graft did not lead to a recurrence of GCT. Non-vascularised fibular strut provided adequate support while the cavity left empty after curettage did not collapse and there was good knee function.

## Introduction

Giant cell tumour is a common bone tumour accounting for 5% of all primary bone tumours. Although the mortality rate associated with the disease is low, the tumour is locally aggressive and has a high tendency to recur^1^. With the advancement in treatment options, the recurrence rate associated with GCT has fallen from an excess of 40% to less than 20% with extended curettage and use of adjuvants^2-4^.

The cavity left behind following the curettage is commonly filled with a bone graft or bone cement. Studies in literature^5,6^ had reported higher recurrence rates when iliac crest bone graft was used to fill the cavity. Bone cement is an inert material and does not get incorporated or remodelled along the lines of stress.

In this study, we postulated that leaving the cavity empty following curettage reduced the recurrence rates while the cavity did not collapse and the cortical struts used to support the empty cavity were incorporated with the host cortex.

## Materials and Methods

Ten consecutive cases of biopsy-proven GCT admitted in our unit between 2015 and 2017 were enrolled in the study. Primary GCT, recurrent GCT and GCT with pathological fractures were all included. Informed written consent was obtained from all the patients. MRI was done to confirm the intramedullary extent of the tumour and possible soft tissue extension. CT chest was done to rule out pulmonary metastasis. All the patients were available for a final follow-up. Ethical clearance was obtained from the Institutional Ethical Committee.

All the cases in our study were treated in the same manner. A creatinine clearance of 60ml per minute was taken as the minimum value for the administration of 4mg of zoledronic acid as per FDA standards. Zoledronic acid was administered in 100ml normal saline over 15 minutes after adequate fluid preloading.

Extended curettage was done three weeks after the first dose of zoledronic acid. The surgery was performed under tourniquet control. The cavity was well visualised, and a curettage of the lesion was done. Power burrs were used to enhance surgical clearance. The cavity was thoroughly irrigated at the end of the procedure to wash away the tumour cells. The cavity was then treated with 3% hydrogen peroxide for three minutes. A total of three hydrogen peroxide washes were given.

The dimensions of the cavity were measured to calculate the length of fibula needed to be resected. Proximal tibia cavities were generally supported by two struts, one mediolateral and one superoinferior strut. Distal femur cavities were given an additional anteroposterior strut when the tumour involved a large portion of the posterior femoral condyle. The measured length of the fibula was resected using a posterolateral approach. The ipsilateral fibula was harvested in lesions involving the distal femur while the contralateral fibula was used in proximal tibia lesions. This protocol was followed to prevent further compromise in the stability of the leg when a fibular defect was made on the same side as the tibial cavity. The fibula was harvested sparing the proximal and distal 8cm.

**Fig. 1:**
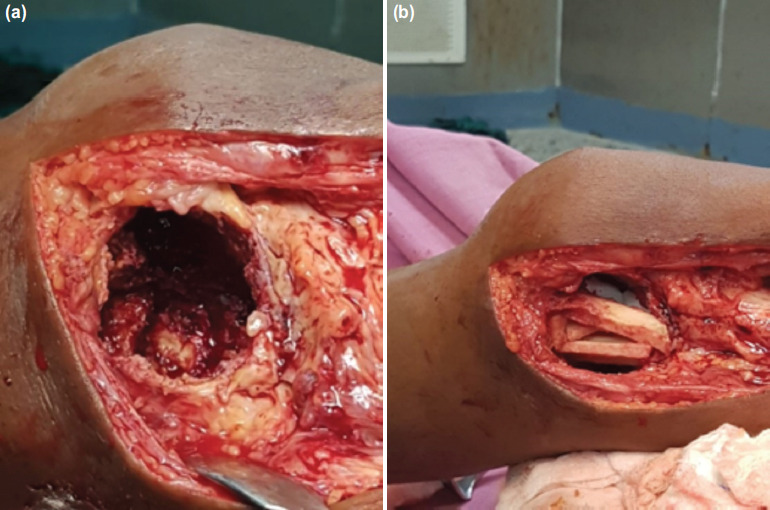
(a) Cavity after curettage (b) Cavity supported with fibula cortical struts.

**Fig. 2:**
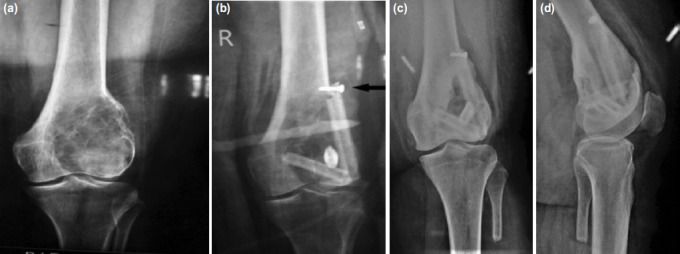
(a) Giant cell tumour involving the lateral femoral condyle in 24-year-old. (b) Cavity was supported with three struts after curettage. Arrow indicates a 3.5mm cortical screw used to secure the superoinferior strut to the lateral femoral cortex. (c,d) Good consolidation of the graft at two years post-operation.

The resected piece of fibula was split into multiple struts. The mediolateral strut was placed first. The superoinferior strut was positioned over the above strut and hitched against the cortex proximally or distally. This construct provided enough support to the cavity (Fig. 1). In one case, the fibular graft was secured to the host cortex using a 3.5mm cortical screw (Fig. 2). The breadth of the graft was more than half the width of the tibial plateau to prevent point loading of the graft.

Patients were given above-knee casts in the immediate postoperative period. Three doses of antibiotics were given. Suture removal was done on the 12th post-operative day. The patients were kept non-weight bearing with above-knee casts. The first follow-up visit was at three weeks post-surgery, during which the second dose of zoledronic acid was given. The final dose of zoledronic acid was given after another six weeks.

Routine radiographs were taken at six weeks, twelve weeks, three months, six months, one year; and at six-month-intervals thereafter. MRI was taken two years post-operative to detect recurrences. The decision to discontinue plaster immobilisation and commence knee mobilisation and weight-bearing was individualised for every patient depending on the consolidation of the graft. The patients were evaluated at six-month intervals using the Musculoskeletal Tumour Society (MSTS) score.

## Results

The average age of the patient in the study was 30 years (range 18 - 39 years). There were five men and five women (sex ratio - one: one). The distal femur (five) and proximal tibia (five) were equally involved in our study. Eight patients had a primary giant cell tumour while two patients had a recurrent giant cell tumour. The primary tumours were staged radiologically using the Campanacci grading. There were five cases of Grade II tumour and three cases of Grade III tumour. All patients were available for the final follow-up. The longest follow-up was 3.5 years, while the shortest follow-up was for 2.5 years. No recurrence was seen in any of the patients as confirmed with MRI scans two years after the surgery. Radiographs revealed good consolidation of the fibular struts and gradual filling up of the cavity. The joint space was maintained in all cases.

Patients were immobilised post-operatively in above-knee casts. The average period of immobilisation was 10 weeks (4 - 16 weeks). The patients were started on toe-touch weight-bearing at the time of plaster removal. The average time taken to start full weight-bearing was 18 weeks (12 - 22 weeks). Four patients regained a knee flexion of more than 90° while six patients had a knee flexion between 60º and 90º. The mean MSTS score of our study was 92%. Two patients had extensor hallucis longus (EHL) weakness on the side of fibular resection which eventually improved.

## Discussion

Ten cases were included in our study, as outlined in Table I. We had eight cases of the primary tumour and two cases of recurrent tumour. Of the two recurrent cases, the first case had a recurrence one year after curettage and cementation. The second case had a recurrence ten years after the first surgery, which was managed with curettage and cementation followed by a second recurrence two years later. The recurrent tumours were all operated primarily at other centres. The use of adjuvant therapy was not documented in both cases. A radical surgery like wide resection offered no significant additional disease control over extended curettage in the management of recurrent GCT. Steyern *et al*^7^ did not find any significant difference in recurrence rate while managing primary and recurrent tumours with extended curettage.

**Table I:** Master chart summarising the findings of the study.

S. No	Age/ Sex	Diagnosis	Campanacci Grading	Period of immobilisation	Time to full weight bearing	Duration of follow-up	Knee flexion	MSTS Score at final follow-up	Complications
1.	35 M	Primary GCT proximal tibia – left side	III	16 weeks	22 weeks	3 years 6 months	120°	29	Nil
2.	34 M	Primary GCT distal femur – left side	II	16 weeks	24 weeks	3 years 6 months	6°	26	Nil
3.	28 M	Primary GCT distal femur – Right side	III	10 weeks	16 weeks	3 years 3 months	75°	28	Nil
4.	34 F	Primary GCT distal femur with pathological fracture-Right side	III	14 weeks	20 weeks	3 years	60º	27	Nil
5.	28 F	Primary GCT proximal tibia with pathological fracture – Right side	II	10 weeks	18 weeks	3 years	60º	26	Nil
6.	18 F	Primary GCT proximal tibia – Right side	II	10 weeks	16 weeks	3 years	120º	29	Nil
7.	38 M	Recurrent GCT proximal tibia – Right side	-	4 weeks	12 weeks	2 years 10 months	75º	27	Nil
8.	23 F	Recurrent GCT proximal tibia – Right side	-	6 weeks	18 weeks	2 years 9 months	110º	29	EHL Weakness
9.	39 M	Primary GCT distal femur – Right side	II	8 weeks	14 weeks	2 years 6 months	75º	27	EHL Weakness
10.	23 F	Primary GCT distal femur – Left side	II	8 weeks	18 weeks	2 years 6 months	100º	28	Nil

**Fig. 3:**
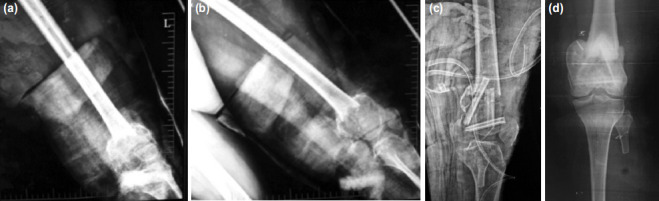
(a,b) Giant cell tumour of distal femur presenting with a pathological fracture. (c) Immediate post-operative radiograph. (d) Two years post-operative radiograph.

The presence of a pathological fracture was not a contraindication for inclusion in our study. Two patients in our series presented with a pathological fracture; one involving the distal femur (Fig. 3), and the other involving the proximal tibia. Intra-operatively, the tumour was found to be well contained within a pseudocapsule, and an intralesional curettage was done with resection of the pseudocapsule. Deheshi *et al*^8^ compared recurrence-free survival and functional outcome after curettage in patients with and without pathologic fracture, with the outcomes being comparable.

Intralesional curettage has emerged as the preferred mode of treatment in GCT, considering the benign nature of the disease and the longer life expectancy of the affected individuals compared to other bone tumours. Systemic adjuvants have supplemented the curettage technique by controlling the micrometastasis. Two commonly used agents included zoledronic acid and denosumab. Zoledronic acid, a third-generation bisphosphonate, acted by promoting the apoptosis of stromal cells, the main neoplastic component in GCT^9^. Several studies had reported low recurrence rates while using zoledronic acid^10-14^.

Tse *et al*^10^ had a recurrence rate of 4% (1 in 24) when zoledronic acid was used as an adjuvant compared to 30% in the group treated without zoledronic acid (6 of 20). The surgical procedure was individualised for each case and varied from curettage with cementation or bone grafting to wide resection. The mean time to recurrence in the control group was 12 months. Gouin *et al*^11^ performed curettage of GCT in 24 patients, using no local adjuvant and filled the cavity with bone cement supplemented with internal fixation. The curettage was supplemented with five doses of post-operative zoledronic acid. There were two recurrences at the end of two years with a third recurrence at the end of five years giving a total recurrence rate of 15%. Yu *et al*^12^ managed 16 cases of GCT of the distal femur with intralesional curettage and cementation supplemented with post-operative oral alendronate. No recurrence was noted at the end of two years. Kundu *et al*^13^ treated 18 cases of GCT with three doses of zoledronic acid pre-operatively. Extended curettage was done two weeks after the last dose, and the cavity was filled with bone graft to eliminate the possible confounding effect of bone cement. They had only one recurrence in the study group (5%) as against four recurrences in the control group (21%). In our series of 10 cases of GCT followed-up for a minimum period of two years, there were no recurrences as confirmed using MRI scans (minimum: 2.5 years; maximum: 3.5 years).

The number and duration of zoledronic acid administered varied in the reported studies. We administered three doses of zoledronic acid at an interval of six weeks. The first dose was given pre-operatively, and two more doses were given after surgery to supplement our extended curettage with hydrogen peroxide. The time interval between administration of the pre-operative dose of zoledronic acid and surgery was 21 days. Nishisho *et al*^14^ advocated a three week waiting period between zoledronic acid and surgery based on in vivo and in vitro studies. Six patients experienced a mild fever within 48 hours of administering zoledronic acid. This is the only notable reaction to the administration of zoledronate. The rise in temperature was benign and settled with antipyretics.

The resected specimens were sent for histopathological examination. The percentage of necrosis of the giant cells in the resected specimens was documented. Pre-operative administration of zoledronic acid had produced more than 50% necrosis in the resected specimens compared to the biopsy tissue. This was consistent with the observations of Cheng *et al* who had a stromal cell necrosis of 54% and giant cell necrosis of 74% while using zoledronic acid^9^.

With the advent of denosumab, promising results had been shown in the management of inoperable or metastatic GCT. However, its superiority over zoledronic acid in conventional limb GCT had not been established^15^. High costs and long duration of treatment before surgery made it less cost-effective. Denosumab promoted new bone formation at the periphery of the tumour, which made the differentiation between normal and pathological tissue difficult during curettage. Neoplastic cells might be left behind the newly formed bone^15^. Denosumab had been associated with a higher incidence of grade 3-4 adverse reactions like osteonecrosis of jaw, hypocalcaemia, anaemia and arthralgia^16^.

The cavity was not filled in our procedure. Bone cavities left behind the following curettage were filled up with blood, which clotted and eventually ossified to form mature bone. Prosser *et al* reported on 166 cases of GCT left empty after curettage, which gradually filled up with new bone that consolidated with time^17^. Iliac crest bone graft was not used to fill the cavity in any patient due to the postulated increase in recurrence reported by several studies when the cavity was filled with bone graft. Gouin *et al*^5^ ascribed an odd’s ratio of 3.9 for recurrence of the tumour when the cavity was filled with cancellous bone graft. Gao *et al* had a threefold increase in recurrence when the bone graft was used compared to bone cement^6^.

Immediate weight bearing could be started when bone cement was used to fill the cavity. Although bone cement allowed for early rehabilitation, it was a biologically inert material and did not remodel along the lines of stress. Another possible complication while using bone cement was the risk of articular cartilage damage on account of variation in modulus of elasticity between bone cement and articular cartilage and the heat generated during cementation^18^. The heat liberated also resulted in a radiolucent zone at the bone cement interface, which could lead on to micromotions between bone and cement resulting in fractures^19^. The sandwich technique was introduced to overcome this potential problem by packing a layer of iliac crest bone graft between the subchondral bone and bone cement. However, this technique introduced bone graft into the cavity, which could enhance the risk of recurrence.

The fibula strut grafts could serve the role of bone cement at the cost of a slight delay in weight-bearing while avoiding the possible joint destruction. These struts had the added advantage of incorporating with the host cortex and remodelling along the lines of stress. The anteroposterior and mediolateral struts were strategically placed over the condyles to ensure coverage over at least two-thirds of the subchondral surface area. Intra-operative varus-valgus stress testing was done to ensure no movement of the graft and collapse of the cavity. Post-operatively patients were immobilised with above-knee slabs till there was adequate new bone formation. By three months, all the cavities showed good consolidation of the graft with adequate support to the subchondral bone. Hypertrophy and incorporation of the fibular graft were noted in all the cases. Krieg *et al* used non-vascularised fibular grafts to bridge 30 cases of tumour cavity post-resection. They concluded that non-vascularised fibular grafts also showed biological activity, evident by their hypertrophy and fracture healing potential through the formation of callus^20^.

Two patients had post-operative extensor hallucis longus weakness on the side of fibular resection. EHL weakness had commonly been reported in literature following resection of long segments of the fibula. Verma *et al*^21^ reported EHL weakness in 43 out of 85 cases of fibular resection (50%). Singhade *et al*^22^ had 10 cases (38%) of EHL weakness following fibular resection. Consistent with the observations of other authors, the weakness was partial and completely recovered within six months in both cases. The distal eight cm of the fibula was preserved to ensure ankle stability. Similarly, the proximal third of the fibula was left intact to protect the common peroneal nerve and knee stability. None of our patients had any functional limitations due to pain over donor site or sensory disturbance.

The average time taken to commence knee mobilisation was 10.2 weeks. The patients were advised to do isometric quadriceps strengthening exercises during this period. The patients were then started on physiotherapy, emphasising on quadriceps strengthening and knee flexion. The prolonged period of knee immobilisation did not affect the postoperative knee range of movement significantly to deter normal activities. Four patients were able to achieve a knee flexion beyond 90º while the other six patients achieved between 60º and 90º of knee flexion.

**Fig. 4:**
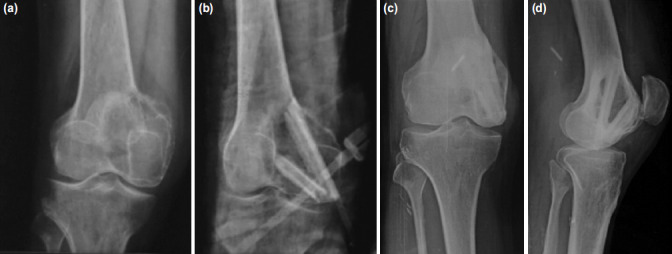
(a) Giant cell tumour of the medial femoral condyle in a 38-year-old. (b) Immediate post-operation radiograph shows cavity supported with two struts. (c,d) Good hypertrophy and incorporation of the graft at two years post-operation.

The average time taken for the patients to fully weight bear without support was 18 weeks (12 - 24 weeks). All the patients were able to resume their pre-surgery work function. Consolidation of the graft was achieved in all the cases. (Fig. 4) The knee was stable, and the alignment achieved intra-operatively was maintained until the final follow-up. The wafer-thin subchondral bone supported only with fibula struts did not collapse, and there was no radiographic evidence of arthritis at the final follow-up.

The mean MSTS score of our series was 92% which is comparable to the results obtained by other surgeons using other modes of treatment. Saibaba *et al*^23^ had an MSTS score of 92% in their series of 36 patients managed with curettage and reconstruction using the sandwich technique. Gao *et al*^6^ had a mean MSTS score of 94.7% in 31 patients managed with curettage and cementation. The major limitations of the study included a short follow-up period, a small sample size and lack of a control group.

## Conclusion

Several methods have been tried by surgeons to decrease the recurrence of GCT. We believed that by not adding cancellous bone graft to the cavity after curettage, with local adjuvant hydrogen peroxide and systemic zoledronic acid to supplement the curettage with power burrs, would decrease the recurrence rates in GCT. Fibula struts provided adequate mechanical stability to the empty cavity. We had no recurrence in any of the cases over a follow-up period of more than two years.
